# Electromagnetic interference safety margins of cardiac implantable electronic devices from electric vehicle charging stations

**DOI:** 10.1093/europace/euag078

**Published:** 2026-05-25

**Authors:** Cecilia Vivarelli, Federica Ricci, Giancarlo Burriesci, Simona D’Agostino, Rosaria Falsaperla, Giovanni Calcagnini, Eugenio Mattei

**Affiliations:** National Center for Artificial Intelligence and Innovative Health Technologies, ISS - Italian National Institute of Health, Viale Regina Elena 229, Rome, Italy; Department of Cardiovascular, Endocrine-Metabolic Diseases and Aging, ISS - Italian National Institute of Health, Viale Regina Elena 229, Rome, Italy; Department of Occupational and Environmental Medicine, Epidemiology and Hygiene, Italian Workers’ Compensation Authority (INIAL), Monteporzio Catone, Rome, Italy; Department of Occupational and Environmental Medicine, Epidemiology and Hygiene, Italian Workers’ Compensation Authority (INIAL), Monteporzio Catone, Rome, Italy; Department of Occupational and Environmental Medicine, Epidemiology and Hygiene, Italian Workers’ Compensation Authority (INIAL), Monteporzio Catone, Rome, Italy; Department of Cardiovascular, Endocrine-Metabolic Diseases and Aging, ISS - Italian National Institute of Health, Viale Regina Elena 229, Rome, Italy; Department of Cardiovascular, Endocrine-Metabolic Diseases and Aging, ISS - Italian National Institute of Health, Viale Regina Elena 229, Rome, Italy

Electric vehicle (EV) charging stations represent an increasingly common source of electromagnetic fields (EMFs) in public and domestic environments, raising questions about possible electromagnetic interference (EMI) with cardiac implantable electronic devices (CIEDs). Although recent clinical studies have not identified malfunctions during EV charging,^1–6^ the actual safety margin between real-world EMF exposure and the minimum interference thresholds defined by international standards remains unknown. This study reports a quantitative assessment of EMF-to-CIED coupling during AC and DC charging under worst case but realistic conditions, using on-site measurements and a sensorized physical twin of a pacemaker/defibrillator. The aim was to determine the induced voltage at the CIED input stage and to quantify the electromagnetic safety margin relative to ISO 14117^7^ requirements.

A measurement campaign was conducted at a public EV charging site equipped with a 22 kW AC (Mode 3) outlet and a 100 kW DC (Mode 4) fast charger. All tests were performed using a Volvo XC40 BEV maintained below 20% state of charge to maximize power draw. Electric and magnetic fields were mapped along charging cables, around cabinet housings, and near the vehicle inlet. A validated physical twin of a pacemaker/ICD implanted in an anthropomorphic saline-filled phantom was used to measure induced voltages in both bipolar and unipolar configurations (*Figure 1*, upper panel).^8^ The phantom allowed reproducible positioning near hotspots, as well as controlled routing of the charging cable across the chest to simulate the most unfavourable scenario. User-contact currents were measured under six realistic touch configurations involving both AC and DC charging hardware.

**Figure 1.**
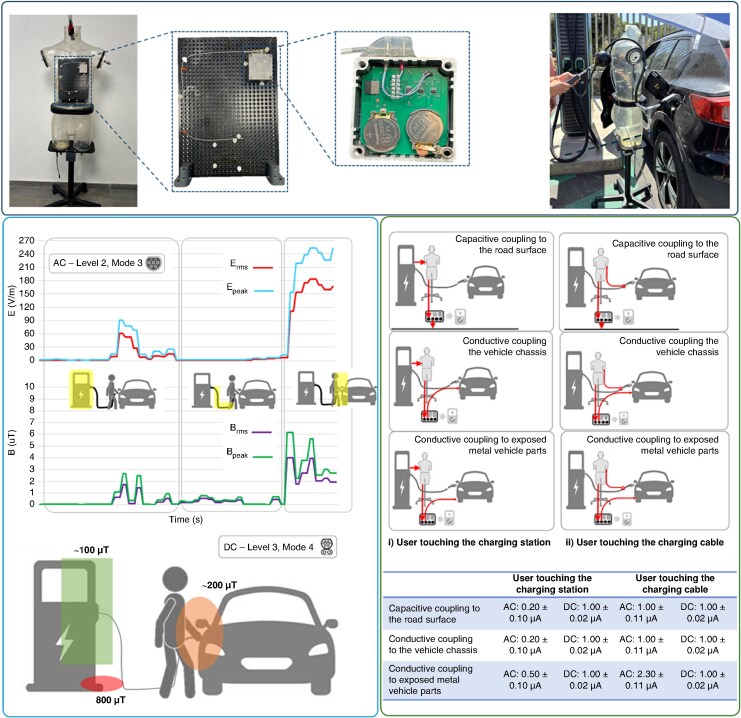
Assessment of the safety margins of cardiac implantable electronic devices from electric vehicle charging stations. Top panels: physical twin of the PM/ICD used to measure the induced voltage and human shaped phantom used to host the physical twin. Bottom left: mapping of the electric (E) and magnetic (B) field while moving from the cabinet along the cable to the vehicle charging inlet, during AC (level 2, mode 3) and DC (level 3, mode 4) charging. Bottom right: User-contact leakage currents measurement setups and results during AC and DC charging.

During AC charging at 11 kW (233 V, 16 A), field mapping revealed the highest EMF levels around the vehicle charging inlet. Peak electric and magnetic fields reached 228 V/m and 5.2 μT, respectively, well below the ICNIRP public exposure limits at 50 Hz (5000 V/m and 100 μT) (*Figure 1*, lower-left panel). Despite testing multiple implant positions and routing the cable across the chest of the phantom, the induced voltage measured by the physical twin remained below 0.04 ± 0.2 mV. These experimental findings are consistent with theoretical predictions based on Faraday’s law: for the measured fields and a 225 cm^2^ induction loop, the induced voltage is expected to remain in the tens to hundreds of microvolts, substantially lower than ISO 14117 immunity thresholds of 2 mV (unipolar) and 0.2 mV (bipolar). From these data, a safety margin of at least 10-fold was demonstrated for the worst-case AC charging scenario.

During DC fast charging at 92 kW (357 V, 258 A), field mapping indicated quasi-static magnetic fields of ∼200 μT near the vehicle inlet and 100 μT around the charging cabinet (*Figure 1*, lower-left panel). A localized hotspot of ∼0.8 mT was detected at the cabinet base, attributable to the internal AC-DC conversion electronics. Although this hotspot exceeds the 0.5 mT action level referenced in Directive 2013/35/EU, it remains below the 1 mT static-field test level specified by ISO 14117 for pacemakers and defibrillators. Again, the physical twin recorded no measurable induced voltage, indicating negligible residual AC ripple coupling to the implant. These data suggest that even at high cable currents, the combination of rapid field decay with distance and compliance with static-field immunities makes interference unlikely for CIED users, consistent with recent clinical observations.

Contact currents were evaluated because 50–60 Hz leakage currents in the tens or hundreds of microamperes are known to trigger CIED oversensing or pacing inhibition under specific conditions [^9,10^]. Across six test scenarios involving contact with the charging station or cable, and simulating capacitive paths to ground or conductive paths to the vehicle chassis or exposed metal components, currents remained in the microampere range, with a maximum of 2.3 μA during AC charging and 1 μA during DC charging (*Figure 1*, lower-right panel). In all cases, the induced voltage at the CIED input stage remained below the resolution threshold of the physical twin, indicating that contact currents generated during EV plug-in and plug-out operations are at least one order of magnitude too small to cause device interference.

These findings provide an experimental and quantitative basis to support the clinical evidence that EV charging systems are safe for users with CIEDs. Prior *in vivo* studies involving up to 350 kW chargers reported no clinically relevant EMI even when patients held charging cables directly over the device pocket. However, such studies do not quantify the safety margin. Our results now demonstrate that EMF exposure levels during AC charging fall well below the thresholds for induced-voltage–based interference, and that DC charging, although generating static magnetic fields that may approach the ISO 14117 static-field immunity level (1 mT), produces field peaks that are confined to localized hotspots unlikely to coincide with the position of an implanted device during routine charging behaviour, and can therefore be considered safe.

An important implication of the present findings is that quantifying the induced voltage at the CIED input stage provides a more robust basis for safety assessment than purely event-based clinical observation. As EV charging infrastructure evolves towards increasingly high-power levels, a margin-based framework enables extrapolation to conditions not yet covered by clinical studies. For example, although high-power DC charging up to 350 kW may produce localized fields exceeding those mapped in this study, the rapid spatial decay observed around the cabinet suggests that even stronger hotspots will remain limited to very specific positions that are not typically occupied by the user during charging. Additionally, since the dominant coupling mechanisms for AC charging scale linearly with electric and magnetic field amplitude, the demonstrated 10-fold safety margin indicates that modest increases in AC power delivery would not approach interference thresholds.

By integrating field mapping, induced-voltage measurement, and contact-current analysis using a reproducible physical model of a CIED implantation, this study provides quantitative reassurance for clinicians, patients, and infrastructure operators. Under realistic worst-case conditions, both AC and DC EV charging systems exhibited exposures far below those required to produce oversensing, pacing inhibition, inappropriate ICD therapy, or asynchronous pacing mode activation. Routine use of public and private EV charging infrastructure can therefore be considered safe for patients with contemporary CIEDs.

## Study limitations

The proposed model does not represent leadless devices or non-transvenous defibrillators (EV-ICDs and S-ICDs), which limits the extension of our findings to these technologies.

## Data Availability

The data underlying this article will be shared on reasonable request to the corresponding author.
